# Gut-Brain Axis in the Early Postnatal Years of Life: A Developmental Perspective

**DOI:** 10.3389/fnint.2020.00044

**Published:** 2020-08-05

**Authors:** Ankita Jena, Carlos A. Montoya, Jane A. Mullaney, Ryan N. Dilger, Wayne Young, Warren C. McNabb, Nicole C. Roy

**Affiliations:** ^1^School of Food & Advanced Technology, College of Sciences, Massey University, Palmerston North, New Zealand; ^2^The Riddet Institute, Massey University, Palmerston North, New Zealand; ^3^Food Nutrition & Health, Grasslands Research Centre, AgResearch, Palmerston North, New Zealand; ^4^High-Value Nutrition National Science Challenge, Auckland, New Zealand; ^5^Department of Animal Sciences, University of Illinois at Urbana-Champaign, Urbana, IL, United States; ^6^Liggins Institute, The University of Auckland, Auckland, New Zealand; ^7^Department of Human Nutrition, University of Otago, Dunedin, New Zealand

**Keywords:** gut-brain axis, brain, gastrointestinal tract, postnatal development, cognition, metabolites, microbiota

## Abstract

Emerging evidence suggests that alterations in the development of the gastrointestinal (GI) tract during the early postnatal period can influence brain development and vice-versa. It is increasingly recognized that communication between the GI tract and brain is mainly driven by neural, endocrine, immune, and metabolic mediators, collectively called the gut-brain axis (GBA). Changes in the GBA mediators occur in response to the developmental changes in the body during this period. This review provides an overview of major developmental events in the GI tract and brain in the early postnatal period and their parallel developmental trajectories under physiological conditions. Current knowledge of GBA mediators in context to brain function and behavioral outcomes and their synthesis and metabolism (site, timing, etc.) is discussed. This review also presents hypotheses on the role of the GBA mediators in response to the parallel development of the GI tract and brain in infants.

## Introduction

The early years of childhood form the basis for physical, metabolic, emotional, cognitive, and social development and have a lasting impact on adult life. Although development starts *in utero*, the developmental events from birth up to 2–3 years of life are equally crucial. This period of life is termed the early postnatal period, where infants undergo rapid developmental maturation in a intrauterine-independent environment. Environmental factors (e.g., diet and early life experiences) are crucial determinants of postnatal development, lifelong health, and wellness.

There is a rapid brain development (e.g., synaptogenesis and myelination) (Knickmeyer et al., 2008) and establishment of cognitive behavioral outcomes in the first 2 years of life (Nelson et al., 2007). The GI tract also undergoes profound growth, morphological changes, and functional maturation, including the establishment of a stable GI microbiota (Xu, 1996; Koenig et al., 2011). Other systems, such as the immune, nervous, skeletal, and circulatory systems, also continue to develop in the early postnatal years of life (Sgarbieri and Pacheco, 2017).

The development phases of the GI tract and the brain are interdependent and occur in a parallel timeline (Carlson et al., 2018; Gao et al., 2019), but are not necessarily synchronous. The developmental interdependency between the GI tract and brain can be attributed to the GBA; a complex, bidirectional communication, incorporating neural, endocrine, immune, and metabolic mediators. The GBA is increasingly recognized as having a role both in physiological and pathological conditions. However, the development of the communication between the GI tract and brain via the GBA remains poorly understood, and more research is required to define better strategies to improve cognitive outcomes, particularly in the early postnatal period.

This review provides an overview of major developmental events in the brain and GI tract in the early postnatal period and their parallel developmental trajectories under physiological conditions. Current knowledge of GBA mediators in context to brain function and behavioral outcomes and their synthesis and metabolism (site, timing, etc.) is discussed. Evidence and hypothesis on GBA mediator’s development in the early postnatal period are also provided.

## Early Life Brain Development

The development of the brain is an organized, predetermined, and highly dynamic multistep process. It begins *in utero* following fertilization and continues postnatally into adolescence in humans (Gibb and Kovalchuk, 2018). During the early postnatal period, brain architecture is shaped and the foundation is set for perceptual, cognitive, and emotional abilities (Paterson et al., 2006). It is increasingly recognized as crucial for the establishment of cognitive and behavioral abilities that last a lifetime (Nelson et al., 2007). Recently, an emphasis has been given to the first 1,000 days, as an opportunity to influence cognitive outcomes in the child (Cusick and Georgieff, 2016). Studies elucidating brain development over this period are vital for research, clinical, educational, and social outcomes. For instance, data on brain development may be relevant for early diagnosis of behavioral disorders like autism (Keehn et al., 2013; Wolff et al., 2015).

The critical brain developmental events include neurulation, neurogenesis, gliogenesis, neural migration, synaptogenesis, myelination, and regressive events like apoptosis and synapse pruning [see reviews by Andersen (2003); Tau and Peterson (2009); Davis (2018)]. In the prenatal period, the development of the brain is mostly influenced by genetic determinants, but in the early postnatal period environmental factors take precedence. Hence, brain developmental events in the early postnatal period are of particular importance, as less favorable environmental conditions can compromise the foundation of brain development, and can have adverse impacts on later stages of life (McCrory et al., 2010).

In the following section, cellular, structural, and functional development of the brain in the early postnatal period are discussed.

### Postnatal Development

In the postnatal period, neurogenesis (formation of neurons) continues to a limited degree in the olfactory bulb (Bergmann et al., 2012) and hippocampal dentate gyrus throughout life (Boldrini et al., 2018). Unlike neurogenesis, gliogenesis (formation of glia) peaks during the first year of life and continues until adolescence (Semple et al., 2013; Reemst et al., 2016; Allswede and Cannon, 2018). Glia has three significant cell subtypes within the brain, namely microglia, astrocytes, and oligodendrocytes, each with different developmental timelines. The microglia regulates neurogenesis, and synaptic refinement (c.f., section “Immune Mediators”) astrocytes support formation and plasticity of the synapse while the oligodendrocytes form myelin (Eroglu and Barres, 2010). The proliferation of microglia peaks in the first 2 weeks after birth and continues until the first month after birth (Budday et al., 2015). The proliferation of astrocytes and oligodendrocytes peak before birth and continue until 15 months of age and adulthood, respectively (Allswede and Cannon, 2018; Davis, 2018). Apoptosis of neuronal cells is largely completed *in utero*, however, apoptosis of the glial cell population continues to occur in the first few months of after birth (Tau and Peterson, 2009; Stiles and Jernigan, 2010). Oligodendrocytes undergo apoptosis to control myelin production during the initial stage of myelination (Caprariello et al., 2015).

Synaptogenesis (formation of the synapse) begins *in utero* but peaks across most of the regions of the brain in the early years of postnatal life (Huttenlocher and Dabholkar, 1997). Synaptogenesis peaks at different times in different regions of the brain, such as in the areas of the cerebral cortex where heterogeneity in synaptogenesis is well documented (Huttenlocher and Dabholkar, 1997). The infant’s brain has almost double the number of synapses compared to the adult brain, and their abundance is reduced by the process of synaptic pruning, which is pronounced during the period of childhood to adolescence (Huttenlocher, 1979; Huttenlocher and Dabholkar, 1997). Together the formation and retraction of synapses shape the neural connections in the brain.

The cerebral cortex is divided into three functionally distinct areas, namely, sensory areas (e.g., visual cortex and auditory cortex), motor areas (e.g., motor cortex), and association areas (e.g., prefrontal cortex). Synaptogenesis in the visual cortex (present in the occipital lobe) peaks at around 6 months of age (Huttenlocher, 1999), whereas in the auditory cortex (temporal lobe) it peaks around 3 months of age, and in the prefrontal cortex (present in the frontal lobe) around 3 years of age (Huttenlocher and Dabholkar, 1997). Hence, this developmental pattern indicates that synaptogenesis peaks first in the sensory and later in the association areas, from a posterior to an anterior direction (Huttenlocher and Dabholkar, 1997; Giedd et al., 1999). The communication across synapses is facilitated by neurotransmitters (c.f., section “Neurotransmitters”) whose abundance increases concomitantly with synaptogenesis (Herlenius and Lagercrantz, 2004).

Myelination is a critical cellular event for the development of the brain, particularly for enhanced neuronal activity and communication. This process consists of the wrapping of axons of neurons with a myelin sheath. Myelination begins in the prenatal period, peaks during the first 3 years of life and continues until the second and third decade years of life (Giedd et al., 1999). Like synaptogenesis, myelination occurs first in the sensory areas followed by association areas of the brain from a posterior to an anterior direction (Volpe, 2000; Barkovich, 2005). Hence, the developmental pattern of synaptogenesis and myelination is indicative of areas with functions that are critical in early life, thus necessitating an earlier requirement for maturation (Huttenlocher and Dabholkar, 1997; Barkovich, 2005).

The brain undergoes significant structural development in the first 2 years of life (Casey et al., 2000). At birth, the total brain volume is 36% of an adult brain, and it reaches around 70% by the first year of age, and 80% by the second year (Knickmeyer et al., 2008). The cortical volume also increases by 88% in the first year and 15% in the second year (Knickmeyer et al., 2008). Cortical volume is determined by the cortical thickness and surface area, and these determinants also change in the first 2 years of life. The increase in cortical thickness and surface area is 31 and 76.4% in the first year of life, and 4.3 and 22.5% in the second year (Lyall et al., 2015). Regional differences in cortical thickness and surface areas are also observed (Shaw et al., 2008; Lyall et al., 2015; Remer et al., 2017). The volume of thalamus and amygdala increases by 130 and 14% in the first and second year, respectively (Knickmeyer et al., 2008). The hippocampus grows slowly in the first year but increases rapidly in the second year, likely linked to the increasing complexity of spatial working memory and path integration when a 2 years child becomes more mobile (Wolbers et al., 2007; Gilmore et al., 2012).

Concurrent with a rapid cellular and structural brain growth is an equally rapid development of the brain functions in the first years of postnatal life (Gilmore et al., 2012). The brain’s functional networks are present *in utero*, but continue to develop in the early postnatal period (Gao et al., 2015): primary sensory-motor and auditory networks are the first to develop, followed by visual, attention, and default mode networks, and finally, the executive control networks begin to emerge (Gao et al., 2015). Different functional networks are activated during different cognitive tasks performed by infants, such as distinguishing different voices, recognizing faces, object permanence, etc. (Paterson et al., 2006).

Changes in both the structural and functional networks of the brain contribute to the development of cognitive abilities (e.g., perception, memory) in the first years after the birth of infants (Gilmore et al., 2018). These developmental events are mainly affected by external factors (diet and early life experiences) (Nelson et al., 2007; Deoni et al., 2018). Any positive and negative alterations of these external factors can either enhance or compromise the development of the brain.

Within the body, the early life development of the brain is co-dependent on the development and appropriate functioning of many organs. It is recognized that the GI tract plays one of the most significant roles in shaping the development of the brain.

## Early Life Gastrointestinal Tract Development

*In utero*, the fetus gets nutrients from the maternal blood via the placenta (Salafia et al., 2007) but after birth, the infant begins enteral nutrition with the uptake of breast-milk (Sangild et al., 2000). This shift from parenteral to enteral nutrition requires a developed GI tract before birth (Sangild et al., 2000). At birth, the tube is fully formed with the required motility functionality to ensure the survival of infant on mother’s breast-milk, independent of placental nutrition (Grand et al., 1976). Details of the GI tract developmental events in the prenatal period have been reviewed elsewhere (Grand et al., 1976; Montgomery et al., 1999; Dimmitt et al., 2018). The GI tract continues to mature in structure and function postnatally, and early life foods are one of its crucial determinants. For instance, mother’s milk and increasingly complex foods after weaning influence the maturation of GI tract (Kelly et al., 1991; Zhang et al., 1998; Jensen et al., 2001) to digests food, absorbs nutrients, and deliver nutrients to the body’s cells for growth development, and maintenance.

### Postnatal Development

The GI tract cellular features are primarily established prenatally followed by structural and functional maturation postnatally in response to early life food (breast-milk and/or formula) (Zhang et al., 1998; Jensen et al., 2001). The structural maturation of the GI tract includes changes in terms of size and anatomical features. Postnatally, the esophagus, stomach, and small and large intestines continue to grow in size (Weaver et al., 1991; Xu, 1996). The postnatal period is also marked by a decline in epithelial permeability (Jakoi et al., 1985; Jakobsson et al., 1986; Drozdowski et al., 2010). After birth, the small intestine is permeable to macromolecules (e.g., immunoglobulin G) present in breast-milk (Jakoi et al., 1985). Within the first few days, the small intestine’s permeability to macromolecule is reduced, which results in cessation of macromolecule transport paracellularly (Jakoi et al., 1985). The exact timing of permeability reduction in humans remains unknown, but studies in piglets and rats suggest that barrier closure happens in the first 2 days after birth (Weström et al., 1984), and by postnatal week 3 (Arévalo Sureda et al., 2016), respectively. Villi development is largely completed at birth, whereas a rapid increase in crypt depth and crypt cell proliferation in the small intestine also occurs in the first years of life, increasing the surface area for nutrient absorption (Thompson et al., 1998; Cummins and Thompson, 2002).

Unlike other peripheral organs, the GI tract has a dedicated nervous system called the enteric nervous system (ENS). The regulation and coordination of muscular and secretory activity by the ENS are required for digestion and absorption (Rao and Gershon, 2016). The ENS is embedded along the wall of the GI tract and consists of a network of neurons that mainly resides within two major ganglionated plexuses (Furness, 2012). The myenteric plexus lies in the muscular propria layer, and the submucosal plexus is in the submucosa layer. In mice, the maturation of ENS in terms of neuronal morphology (e.g., dendritic and axonal structure), types of neurons (e.g., cholinergic and nitrergic), neurally mediated motility patterns in different regions of GI tract occurs during the postnatal period, as reviewed by Foong (2016). For detailed information on ENS development, readers are redirected to the extensive compilation by Rao and Gershon (2018).

*In utero*, the GI tract of the fetus is exposed to amniotic fluid, which contains 98% water and 2% protein, sodium, chloride, and CO_2_ (i.e., low nutrient content) (Bonsnes, 1966). Immediately after birth, the infant is introduced to colostrum, which is rich in proteins (e.g., lactoferrin and lactoperoxidase), immunoglobulins, and growth factors (e.g., epidermal growth factor and vascular endothelial growth factor) (Ballard and Morrow, 2013; Godhia and Patel, 2013). The infant GI tract undergoes further functional development to adapt to complex and more diverse nutrient profiles postnatally (Hampson, 1986; Thompson et al., 1998). The activity of the enzymes enterokinase (protein hydrolysis), gastric lipase (lipid hydrolysis), and lactase (carbohydrate hydrolysis) increases gradually after birth (Antonowicz and Lebenthal, 1977; Moreau et al., 1988; Shulman et al., 1998) to facilitate the digestion of complex food structures. Functional maturation of the GI tract in the postnatal period also includes the establishment of the microbiota.

#### Microbial Colonization

The colonization of microbes in the GI tract begins at birth and continues until about 3 years of age when the composition becomes adult-like (Koenig et al., 2011; Yatsunenko et al., 2012). However, the literature suggests the presence of microbes *in utero.* This view arises from the fact that microbes have been detected in the meconium (i.e., the first stool of infant after birth), amniotic fluid, and placenta (Aagaard et al., 2014; Ardissone et al., 2014; Urushiyama et al., 2017; Shi et al., 2018). A study by Ardissone et al. (2014) showed that approximately 61% of the microbial population in meconium was found to be similar to that of the amniotic fluid, suggesting that microbes in the meconium originate by swallowing of amniotic fluid by the fetus (Ardissone et al., 2014). The viability of microbes *in utero* remains debated in the scientific community, and the problem of contamination artifacts is an issue discussed among researchers. However, recent mouse studies showed viable bacteria in the fetal gut, uterus, and placenta, suggesting the possibility of the presence of viable bacteria in a human fetus (Younge et al., 2019). Therefore, more studies on *in utero* colonization are warranted to challenge the accepted sterile womb paradigm.

In the postnatal period, the microbial colonization of the infant GI tract follows a succession of steps. Studies of the GI microbiota in the infant are limited to fecal samples. Stool samples are a proxy for the microbial population of the large intestine but may not represent it accurately. During the first few weeks after birth, the GI microbiota of infants is dominated by facultative anaerobes like members of the *Enterobacteriaceae* family (Palmer et al., 2007; Matsuki et al., 2016; Nagpal et al., 2017), which are likely coming from the mother’s vagina and skin (Palmer et al., 2007; Lozupone et al., 2013). At around 6 months, strict anaerobes, including bacteria of the *Bifidobacterium*, *Clostridium*, and *Bacteroides* genera, dominate the composition (Nagpal et al., 2017). At around 3 years of age, the microbiota profile shows a high degree of resemblance to that of adults (Palmer et al., 2007; Koenig et al., 2011; Yatsunenko et al., 2012) and is represented almost entirely by strict anaerobes like the *Clostridium coccoides* group, *Clostridium leptum* subgroup, and *Prevotella* (Nagpal et al., 2017).

However, the GI microbial community consists not only of bacteria but also include phage, archaea, and fungi. Most studies have focused on bacterial colonization of the GI tract in infants, and much less is known about other kingdoms of life. According to the available knowledge, bacteriophage, mainly of the Caudovirales order and *Microviridae* family, archaea *Methanobrevibacter smithii*, and fungi *Candida albicans* are the most predominant non-bacterial organism in the infant GI tract during the first years of life (Palmer et al., 2007; Smith et al., 2013; Heisel et al., 2015; Lim et al., 2015, 2016; Schei et al., 2017; Ward et al., 2017).

The transition from milk to solid food is one of the influential factors of the colonization process in infants (Fallani et al., 2011; Koenig et al., 2011; Turroni et al., 2012). More studies where the analysis of the bacteria, phage, archaea, and fungi composition and function are needed to fully understand the colonization patterns and their temporal changes during the transition from milk to solid foods.

## Parallel Development Between the GI Tract and Brain

**FIGURE 1:**
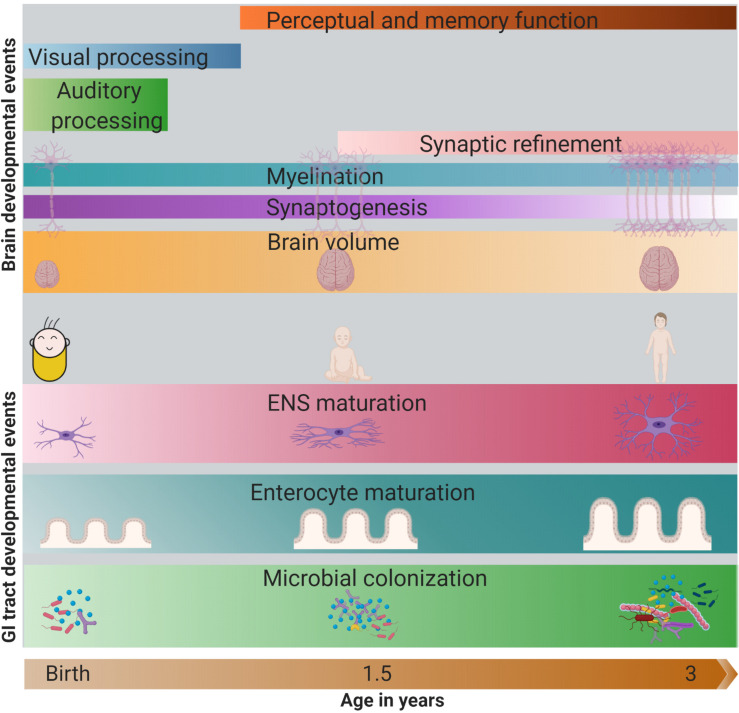
Parallel development of the GI tract and the brain in first 3 years of life. In the GI tract, increase in microbial abundance and diversity, enterocyte maturation (change in crypt and villi structure) and ENS maturation (change in nerve density, type of neurons) occurs rapidly in the first 3 years of life. Meanwhile, brain also develops rapidly, with the change in its volume (peak in the first year), synaptogenesis, myelination, synaptic refinement, and establishment of cognitive abilities like auditory and visual processing, perception, and memory. The darkness of the color represents the intensity/peak of the developmental event. GI, gastrointestinal; ENS, enteric nervous system. Note: The developmental timing of the cellular events may vary across different regions of the brain.

The majority of the development of the GI tract and brain occur in parallel, but their development is asynchronous in terms of attaining peak and maturity. For instance, microbial colonization, tissue structural maturation, and ENS maturation coincide with the refinement and remodeling of brain neural circuits and cognitive development in the first years of life (Figure 1). There is increasing evidence that the colonization of the GI tract by the microbiota appears to have a parallel developmental trajectory to the brain for up to 3 years of age. A study by Carlson et al. (2018) showed that infants with a high relative abundance of *Bacteroides* in their stools had better cognitive performance in terms of receptive language and expressive language. In contrast, infants with a high level of *Faecalibacterium* in their stools had lower cognitive performance (Carlson et al., 2018). Another study in infants showed a positive association of the alpha diversity of the fecal microbiota and the functional connectivity between the supplementary motor area and the inferior parietal lobule (areas associated with cognitive outcomes) of the brain (Gao et al., 2019).

Evidence from rodent studies has also provided insights into the correlation between changes in the GI microbiota and brain function in early postnatal life. Germ-free (GF) mice displayed altered anxiety responses, abnormal motor activities, enhanced stress responses, and memory dysfunction (Sudo et al., 2004; Gareau et al., 2011; Heijtz et al., 2011). Interestingly, when GF mice are conventionalized with microbiota obtained from specific pathogen-free (SPF) mice in adulthood rather than early life, anxiety-like behavior associated with altered synaptic related proteins and neurotransmitter turnover persist (Sudo et al., 2004; Heijtz et al., 2011). These findings suggest that specific changes in brain structure and function cannot be reversed beyond a critical window in the early postnatal period (Sudo et al., 2004; Heijtz et al., 2011).

Additionally, adult GF mice exhibit a decreased production of the neurotransmitter serotonin (5-HT) in the GI tract, as compared to conventionally raised and SPF adult mice (Reigstad et al., 2015; Yano et al., 2015). 5-HT is produced both in the brain and the GI tract (c.f., section “Tryptophan Metabolites”). It is well known that brain-derived 5-HT is associated with mood regulation, learning, and memory (Cowen and Sherwood, 2013; Carhart-Harris and Nutt, 2017), but whether changes in GI-derived 5-HT regulate these brain functions, remains to be confirmed.

A study by Collins et al. (2014) showed that, at 3 days of age, the development of myenteric plexus of the ENS was structurally abnormal in GF mice compared to that of SPF mice. The myenteric plexus showed decreased nerve density and ganglionic size but increased nitrergic neurons in the GF mice (Collins et al., 2014). Whether these functional changes in the GI tract translate into cognitive outcomes, remain unknown, but it is plausible that there is an interdependency between the establishment of the GI microbiota, the ENS and the development of the brain. It is important to note that studies in rodent models may not be reproducible in humans, as there is a marked difference between rodents and humans in terms of the developmental patterns of the GI tract and brain. Rodents are born with a relatively underdeveloped GI tract, and most of the functional development occurs in the postnatal period (Searle, 1995; Drozdowski et al., 2010; Guilloteau et al., 2010). The timing of brain developmental events is also different between humans and rodents (Pressler and Auvin, 2013). The anatomy and physiology of the GI tract, brain growth, and developmental patterns of both organs in piglets share a greater similarity to humans than other non-primate models like rodents (Guilloteau et al., 2010; Mudd and Dilger, 2017).

Most studies of GI and brain development have been mainly focused on the role of the GI microbiota. The GI tract undergoes developmental changes not only in terms of microbiota but also enzyme activity, gastric secretions, small intestinal permeability, and increased surface area for absorption of nutrients (i.e., crypt-villi structural modification) (c.f., section “Postnatal Development”). How these changes in the GI mucosa affect brain outcomes remains mostly unknown. For instance, an increase in the surface area of absorption of nutrients over this period could result in increased availability of nutrients for the host and less for the microbiota. The result could be a profile of different neuroactive metabolites in the GI tract contributing to specific cognitive outcomes. However, no studies have been conducted to relate structural and functional modifications in the GI tract to brain developmental events in the early years of postnatal life.

## Gut-Brain Axis

The GI tract and the brain are connected through a complex network of signaling pathways collectively termed as the GBA (Carabotti et al., 2015). In the last decade, the role of GI microbiota in the GBA has been extensively assessed, and the term has been extended to microbiota-GBA. Here, the term GBA includes the microbiota. The communication between the GI tract and brain is bidirectional and is mediated by neural, endocrine, immune, and metabolic mediators.

**FIGURE 2:**
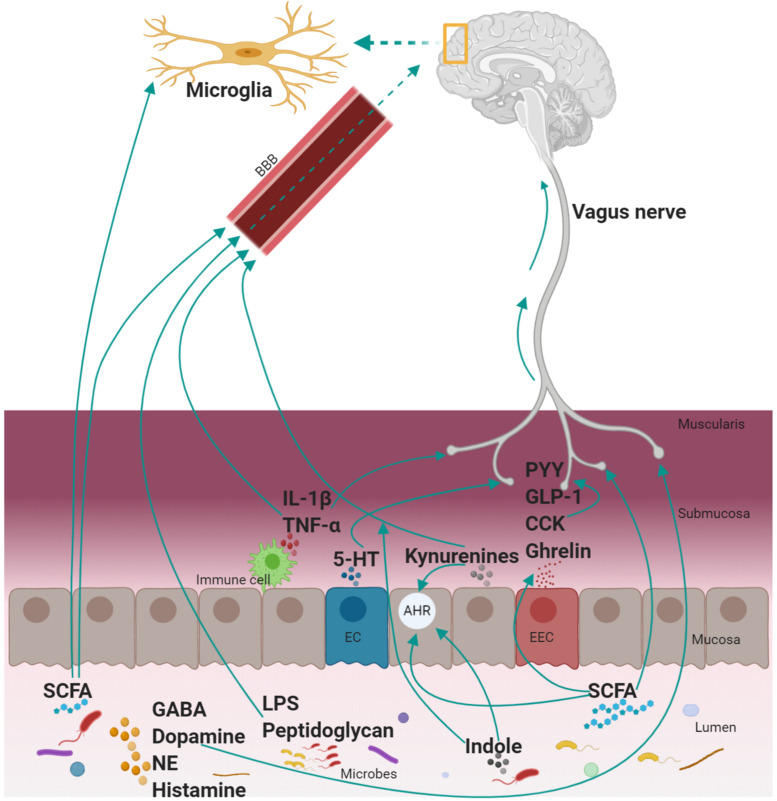
Mechanism of communication between the GI tract and the brain. A myriad of mediators is involved in the complex communication between the GI tract and the brain. These include neural (vagus nerve), endocrine (hormones; PYY, GLP-1, CCK, and ghrelin), immune [cytokines (IL-1β and TNF-α), microglia, microbial antigenic component (LPS, peptidoglycan), and metabolic (TRP metabolites (kynurenines, 5-HT, and indole), SCFA, neurotransmitters (GABA, dopamine, NE, and histamine)] mediators. The mode of action of these mediators is by: activating the vagus nerve or crossing the BBB to communicate with the brain directly. SCFA regulates other mediators (EEC to produce hormones, microglia maturation, AHR activation; an essential receptor for TRP metabolites (produced both by the host and microbiota). GI, gastrointestinal; GLP-1, glucagon-like peptide-1; PYY, peptide YY; CCK, cholecystokinin; TNF-α, tumor necrosis factor-α; IL-β, interleukin-β; GABA, gamma-aminobutyric acid; NE, norepinephrine; SCFA, short-chain fatty acids; EEC, enteroendocrine cells; TRP, tryptophan; LPS, lipopolysaccharides; BBB, blood–brain barrier; AHR, aryl hydrocarbon receptor; EC, enterochromaffin cell; 5-HT, serotonin. Note: Kynurenines include kynurenine and downstream metabolites of the kynurenine pathway and not necessarily all the kynurenines can cross the blood–brain barrier.

The GBA has been studied using top-down and bottom-up approaches. The modulation of the GI functions by the brain (top-down approach) is well established by preclinical and clinical evidence. For instance, modulation of motility, secretion (HCl acid in the stomach, bicarbonates in pancreatic juice, and mucus by goblet cells), and mucosal immune responses in the GI tract are controlled by the brain as reviewed by Rhee et al. (2009). The modulation of brain functions by GI-derived molecules (bottom-up approach) involves different signaling pathways (Figure 2). The importance of the GBA is increasingly recognized both in physiological (e.g., GI homeostasis) and pathological conditions (e.g., mood disorders, obesity, and autism) and have been extensively reviewed in Mayer (2011); Agustí et al. (2018); Liu and Zhu (2018); Martin et al. (2018). However, the understanding of GBA during the co-development of the GI tract and the brain in the early postnatal period is limited.

### Neural Mediators

The vagus nerve (VN) is the longest nerve in vertebrates and innervates many visceral organs like the heart, lungs, and GI tract (Bonaz et al., 2018). It has a vital role in many functions such as digestion, immune responses, heart rate, and controlling mood (Breit et al., 2018). The VN also plays a crucial role in facilitating neural signals between the GI tract and the brain (Bravo et al., 2011). It is the principal component of the parasympathetic nervous system and is composed of 80% afferent and 20% efferent fibers (Bonaz et al., 2018). The afferent fiber carries information from the GI tract to the brain, and the efferent nerve fiber carries information from the brain to the GI tract. The efferent fiber mainly regulates motility and glandular secretion in the GI tract, possibly by interacting with the ENS, mainly by cholinergic activation via nicotinic receptors (Garza et al., 2009; de Jonge, 2013). Over the last decade, the vagal afferent pathways have been increasingly recognized as sensors of hormones, cytokines, and metabolites produced in the GI tract with potential consequences for brain function and behavior. The afferent pathway is also involved in the activation and regulation of the hypothalamic-pituitary adrenal axis, a principal component of the physiological stress system, and a key mediator of the GBA during stress as reviewed by De Weerth (2017).

Vagal afferent fibers are located in all layers of the GI tract but do not cross the mucosal layer outwardly (Wang and Powley, 2007). Thus, they cannot sense the luminal contents directly, but indirectly through the diffusion of microbial metabolites such as short-chain fatty acids (SCFA) (Lal et al., 2001) or via enteroendocrine cells (EEC) (Li et al., 2000). The EEC represent about 1% of epithelial cells and form the largest endocrine organ of the body (Mayer, 2011). These cells are capable of sensing luminal contents, and in response they produce and release molecules (e.g., a variety of hormones and 5-HT) that bind to receptors expressed on afferent endings (Egerod et al., 2012, 2018). A study showed that the administration of *Lactobacillus rhamnosus* improved depression and anxiety-like behavior in mice (Bravo et al., 2011). These effects were not observed in vagotomized mice, suggesting the importance of the VN in delivering improving brain functions in response to a specific bacterium (Bravo et al., 2011).

In the early postnatal life, the VN is not fully functional. Infants are born with the VN only partially myelinated (Porges and Furman, 2011). As discussed before (c.f., Section “Postnatal Development”), nerve myelination continues in the postnatal period, and that also holds for the VN. Development from partially myelinated to fully myelinated VN starts at approximately 24 weeks of gestation and continues until adolescence (Sachis et al., 1982; Porges and Furman, 2011). However, a faster VN myelination rate was observed from 32 weeks of gestation until 6 months after birth (Sachis et al., 1982), suggesting accelerated transmission of signals between the GI tract and brain during this period, likely due to the consumption of breast-milk by infants. Milk is an essential source of long-chain polyunsaturated fatty acids (e.g., docosahexaenoic acid and arachidonic acid), sphingolipids (e.g., sphingomyelin), phospholipids (e.g., phosphatidylcholine), and cholesterol, which are all essential for myelin sheath synthesis and development (Deoni et al., 2018). However, the effects of breast-milk or substitutes on the myelination of the VN is poorly understood.

### Endocrine Mediators

The hormones produced by EEC are essential mediators of the GBA. Ghrelin, glucagon-like peptide (GLP)-1, cholecystokinin and, peptide YY (PYY) are produced and released by EEC in response to the food intake and composition (Egerod et al., 2012; Latorre et al., 2016). These hormones regulate food intake, satiety, gastric emptying, and energy balance by transmitting signals between the GI tract and the brain, reviewed in Raybould (2007); Cong et al. (2010); Holzer and Farzi (2014). Ghrelin is mainly released by the stomach, and it stimulates gastric emptying, regulates appetite, and increases the release of growth hormone (Kojima et al., 1999; Sun et al., 2004). Cholecystokinin and GLP-1 are produced in the small intestine and inhibit gastric emptying and reduces food intake (Liddle, 1997; Holst, 2007). The site of production of PYY is the ileum and the colon, and it decreases gastric motility, improves glucose homeostasis, and induces satiety (De Silva and Bloom, 2012).

Studies have shown that GI hormones also play a crucial role in regulating emotion and mood. For instance, ghrelin reduces anxiety-like and depressive-like symptoms of chronic stress (Lutter et al., 2008), whereas high PYY, mimicking its postprandial plasma concentration, promotes hedonic behavior (Batterham et al., 2007). It remains to be proven that these effects occur in physiological conditions. A variety of GI hormones are produced in normal physiological conditions, and the effect of one hormone is possibly counterbalanced by others. For instance, GLP-1 enhance anxiety-like behavior (Möller et al., 2002; Gulec et al., 2010), whereas GLP-2 could attenuate depression-like behavior (Iwai et al., 2009). These hormones regulate the signaling between the GI tract and the brain, most likely by activating the receptors present in the vagal afferent fiber (Egerod et al., 2018; Okada et al., 2018).

The type of feeding is known to influence the production of GI hormones. Infant fed infant formula during the first 6 months of age had higher ghrelin and lower PYY blood concentrations compared with infants fed breast- milk over the same period (Breij et al., 2017). However, there are no studies that report associations between changes in GI hormones and behavior over the developmental phase of both tissues and in response to feeding types in infants. Additionally, the signals from endocrine hormones may be altered during VN myelination in early postnatal life (c.f., section “Neural Mediators”).

### Immune Mediators

The constituents of the immune system, immune cells and signaling molecules, act as an important intermediary in the GBA. Microglia, the tissue-resident immune cells in the brain, has increasingly been recognized as a significant neuroimmune player of the GBA and in early life brain development (Erny et al., 2015). For instance, the microglia regulates neurogenesis and synaptic refinement (c.f., section “Postnatal Development”) by phagocytosing excess neurons and synapses (Schafer et al., 2012; Cunningham et al., 2013). Regulation of neurogenesis is crucial for ensuring that this process does not exceed neuron’s demand of the developing brain, and ultimately aides in brain organization (Cunningham et al., 2013). Synaptic refinement is essential for shaping the neural circuitry by eliminating the redundant synapses during postnatal brain development (Wu et al., 2015). A study by Erny et al. (2015) showed that the microglia in adult GF mice have abnormal morphology and density, altered cell proportions (e.g., dendrite length), and immature phenotype when compared with SPF mice. These adverse effects were partially rectified when adult GF mice were colonized with complex microbiota, suggesting a role for the microbiota in microglia maturation and function (Erny et al., 2015). It is important to note that the oral administration of a mixture of SCFA (acetate, propionate, and butyrate) (c.f., section “Short-Chain Fatty Acids”) was sufficient to drive the maturation of the microglia in GF mice (Erny et al., 2015). However, the mechanism underlying the maturation of effects of SCFA remains to be determined. Evidence from these studies points out to a relationship between the microbiota and the microglia that could be important in the immune-mediated aspects of the GBA and brain development in the early postnatal life.

The signaling molecules of the immune system (e.g., cytokines) also participate in the GBA, possibly by two mechanisms: binding to VN receptors or transport across the BBB. Evidence shows that the afferent VN fiber has receptors for the cytokine interleukin-1β (Ek et al., 1998). This cytokine is capable of triggering its production and other proinflammatory cytokines that induce neuroinflammation (Shaftel et al., 2007). Tumor necrosis factor-α can cross the BBB (Gutierrez et al., 1993) and results in neuroinflammation and dysfunction in the brain (Seleme et al., 2017). Bacterial peptidoglycan (outermost covering of Gram-positive bacteria) derived from resident commensals could also cross the BBB under physiological conditions, thereby influencing the brain development and the social behavior in 3-day-old mice (Arentsen et al., 2016).

Another study in rats has shown that lipopolysaccharides (LPS), from the surface of Gram-negative bacteria, can also cross the BBB (Vargas-Caraveo et al., 2017). Studies in mice have shown that intraperitoneal injection of LPS resulted in a decrease in novel object exploratory behavior by impairing continuous attention and curiosity toward objects (Haba et al., 2012). LPS can bind to the toll-like receptor 4 expressed on the microglia (Laflamme and Rivest, 2001) and afferent VN (Hosoi et al., 2005). However, the relationship between LPS-driven immune activation and alteration of behavior remains to be established.

The immune system in the early postnatal period undergoes the most rapid and radical changes compared with other systems in the body (Goenka and Kollmann, 2015). Commensal microbiota is essential for driving normal immune stimulation and maturation (Kamada et al., 2013; Olin et al., 2018). In infants, the cells of the innate immune system (e.g., monocytes and macrophages) are mostly developed prenatally, but their functions remain less developed in newborns (Simon et al., 2015). This lower activity could be to avoid unnecessary immune reactions during the period of continuous developmental remodeling (Prabhudas et al., 2011; Franchi et al., 2012). The cells of the adaptive immune system (e.g., B and T cells) are low in number and are functionally immature in infants (Tasker and Marshall-Clarke, 2003; Haines et al., 2009), which is most likely due to limited exposure to antigens required to develop an immune memory (Prabhudas et al., 2011). With the development of immune cells in early life, the level of their secretory products (i.e., cytokines) can also change over time (Corbett et al., 2010). This dynamic nature of immune mediators in the early postnatal life is likely to contribute to the development of the brain and associated behavior.

### Metabolic Mediators

Metabolites are low molecular weight compounds, typically under 1,000 Da, which are reactants, intermediates, or products of enzyme-mediated biochemical reactions (Fanos et al., 2012). Metabolites play essential roles in the GBA and can have either direct or indirect (e.g., interaction with a neural mediator) effects on brain function. Metabolites can be produced either by the host, the GI microbiota, or the interactions in between them. Among various metabolites produced in the body, tryptophan (TRP) metabolites, SCFA, and neurotransmitters are increasingly recognized as potential mediators of the GBA.

#### Tryptophan Metabolites

Tryptophan is an essential amino acid for the synthesis of body proteins, and it is a precursor to several metabolites. Once absorbed, TRP can be metabolized in enterocytes and hepatocytes, thereby reducing its availability to the rest of the body, including the brain (Waclawiková and El Aidy, 2018). TRP is metabolized through different pathways (hydroxylation and kynurenine) in the GI mucosa, producing neuroactive compounds (Bender, 1983) that are of importance for the GBA.

The hydroxylation pathway generates two important metabolites, 5-HT and melatonin that participate in the GBA (Bender, 1983). The neurotransmitter 5-HT is involved in GI functions such as gastric secretion and motility (Gershon and Tack, 2007), and in the brain it regulates mood and is involved in cognitive and behavioral functions (Cowen and Sherwood, 2013; Carhart-Harris and Nutt, 2017). About 95% of total 5-HT in the body is synthesized by enterochromaffin cell, a subtype of EEC, and 5% is synthesized in the central nervous system (Gershon and Tack, 2007). So far, there is no evidence for the production of 5-HT by the GI microbiota, but studies have shown that microbiota mediates 5-HT synthesis in EEC, which could account for up to 50% of GI-derived 5-HT (Reigstad et al., 2015; Yano et al., 2015).

There is no evidence supporting that 5-HT produced in the GI tract can cross the BBB. Nakatani et al. (2008) showed that brain-derived 5-HT could cross the BBB to reach the peripherical circulation in rats. Interestingly, microbes in the GI tract have shown to influence the brain 5-HT level in a mouse model (Clarke et al., 2013). More studies are required to evaluate the bi-directional transport of 5-HT across the BBB and the potential regulatory role by the GI microbiota. Recently, studies have shown that certain commensal microbes and probiotic strains can uptake luminal 5-HT via specific transporters, which in turn can influence the microbial colonization of the GI tract (Lyte and Brown, 2018; Fung et al., 2019). By linking these findings, it could be inferred that the GI microbiota both requires 5-HT produced in the GI tract and regulates the concentration of 5-HT both in the GI tract and brain. Hence, the role of microbiota in the host serotonergic system warrants further attention.

**FIGURE 3:**
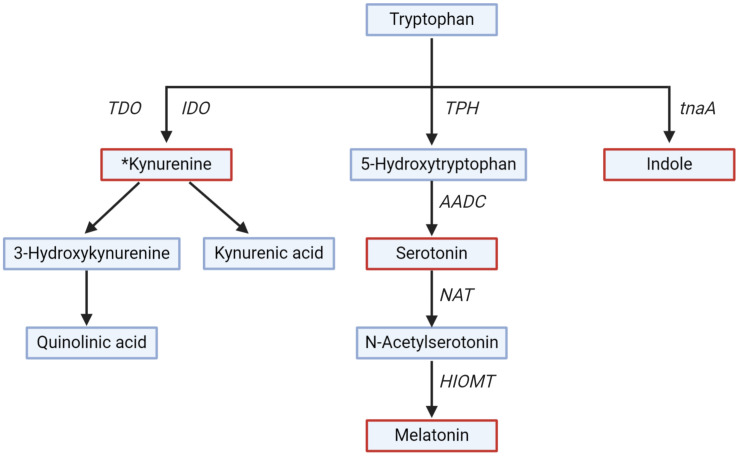
Tryptophan metabolism along different pathways. Key metabolites (serotonin, melatonin, kynurenine, and indole) are in red. Rate-limiting enzymes shown are Trpytophan-2,3-dioxygenase (TDO), indoleamine-2,3-dioxygenase (IDO), tryptophan hydroxylase (TPH), Aromatic L-amino acid decarboxylase (AADC), *N*-acetyl transferase (NAT), hydroxyindole-*O*-methyl transferase (HIOMT), tryptophanase (tnaA). *All the downstream metabolites and enzymes of the kynurenine pathway have not been shown for simplicity.

TRP is metabolized to 5-HT in a two-step process (Figure 3). TRP hydroxylase (TPH), a rate-limiting enzyme in the biosynthesis of 5-HT, exists in two isoforms in the GI tract (TPH1) and the brain (TPH2) (Bender, 1983; Badawy, 2019). The conversion of 5-HT to melatonin is another two-step process and is catalyzed by two limiting enzymes: *N*-acetyl transferase and hydroxyindole-*O*-methyltransferase (Bender, 1983; Zagajewski et al., 2012). Melatonin is produced both in the GI mucosa and the pineal gland (Zagajewski et al., 2012). Melatonin regulates circadian rhythms of behavior, physiology, and sleep patterns, and also regulates GI motility (Richard et al., 2009).

The kynurenine pathway is gaining interest due to the role of kynurenine and downstream metabolites (collectively called kynurenines) on the GI tract and brain functions, and thus on GBA signaling (Cervenka et al., 2017). The kynurenine pathway is responsible for around 90% of TRP degradation (Badawy, 2017). Kynurenine is produced from TRP by the action of TRP-2,3-dioxygenase and indoleamine-2,3-dioxygenase (IDO) (Platten et al., 2019). Kynurenine is further metabolized into downstream metabolites, of which kynurenic acid, 3-hydroxykynurenine, and quinolinic acid are of particular interest for their neuroactive effect on the brain (Badawy, 2017). The metabolite kynurenic acid has a neuroprotective effect, while 3-hydroxykynurenine and quinolinic acid have a neurotoxic effect (Schwarcz and Stone, 2017). The enzyme TRP-2,3-dioxygenase is expressed in the liver, and IDO is widespread in numerous tissues, including the GI tract and the brain (Le Floc’h et al., 2011). The activity of IDO is regulated by proinflammatory cytokines (e.g., interferon-γ) released by toll-like receptor activation (Mahanonda et al., 2007), suggesting that the kynurenine pathway is more active in periods of immune activation or pathological conditions (Clarke et al., 2012). Unlike 5-HT, kynurenine, and 3-hydroxykynurenine produced in the GI tract can cross the BBB and can be further metabolized in the brain (Fukui et al., 1991).

An increase in TRP metabolism along the kynurenine pathway can result in a reduced availability of TRP for 5-HT synthesis and increased production of harmful kynurenine metabolites in the brain, contributing to mood disorder (Maes et al., 2011). This may also imply decreased melatonin levels which are associated with circadian malfunctioning and can increase the risk of mood disorders (Quera-Salva et al., 2011). Interestingly, melatonin appears to promote the expression of IDO, suggesting a negative feedback loop through which melatonin regulates the balance between kynurenine and 5-HT pathways (Li et al., 2017).

The GI microbes can also metabolize TRP (Wikoff et al., 2009; Zheng et al., 2011; Waclawiková and El Aidy, 2018). The primary metabolite produced by microbial metabolism of TRP is indole, which is catalyzed by the enzyme tryptophanase (Jaglin et al., 2018; Waclawiková and El Aidy, 2018). Recently, Jaglin et al. (2018) have shown that administration of indole directly in the rat’s cecum, where microbes metabolizing TRP to indole are highly abundant, was associated with decreased motor activity and anxiety-like behavior. However, the effect of indole on the human brain and behavior has not been studied yet.

It is important to note that TRP metabolites: kynurenine, kynurenic acid, indole, and indole- derivatives are important ligands for aryl hydrocarbon receptor (AHR) (DiNatale et al., 2010; Mezrich et al., 2010; Jin et al., 2014). The AHR is a cytoplasmic ligand-induced receptor, which is ubiquitously expressed on almost all tissues (Yamamoto et al., 2004) and contributes to immune homeostasis by having an antimicrobial and anti-inflammatory effect (Zelante et al., 2013, 2014). For instance, lactobacilli utilize TRP to produce indole-3-aldehyde, an AHR ligand, which has shown to activate innate lymphoid cells that provide mucosal resistance against the pathogen *C. albicans* (Zelante et al., 2013). Interestingly, microbial metabolites such as SCFA were found to regulate AHR and its target genes in the intestine, which in turn influence the microbial composition, highlighting the bi-directional communication of AHR and the GI microbiota (Korecka et al., 2016). Evidence on the role of AHR in brain development and function is limited. A study by Latchney et al. (2013) showed altered hippocampus neurogenesis and contextual fear memory in AHR deficient adult mice, suggesting a role of AHR in brain development. Whether the regulation of neurodevelopment by AHR is due to TRP metabolites is yet to be proven.

The combined increase in surface area for nutrient absorption (Thompson et al., 1998) and diversity of the commensal microbiota (Nagpal et al., 2017) during the maturation of the GI tract in the early postnatal period, means that more TRP is absorbed and/or more TRP metabolites are produced and released in the peripheral circulation. However, the impact of GI tract maturation on TRP metabolism in the early postnatal life is poorly understood. Interestingly, a study in infants showed that cereals enriched with TRP increased plasma concentrations of melatonin and improved sleep quality (Cubero et al., 2009). As the sleep-wake cycle is controlled by TRP-derived melatonin (Brown, 1994) and more melatonin levels resulted in better sleep (Cubero et al., 2009). This evidence could be indicative of more TRP metabolism through the hydroxylation pathway than other pathways. The role of the TRP pathways and resulting neuroactive metabolites in brain development and function in early postnatal life is a fertile area of research.

#### Short-Chain Fatty Acids

The organic acids SCFA are saturated fatty acids with a chain length from one to six carbon atoms. They are the primary end-products of bacterial fermentation and are produced in the GI tract depending on the content of dietary (e.g., fiber) (Bergman, 1990), and non-dietary components (e.g., mucins) (Hoskins and Boulding, 1981; Montoya et al., 2017). The most abundant SCFA produced in the human GI lumen are acetate, butyrate, and propionate (Dalile et al., 2019). The majority of SCFA produced are absorbed (Ruppin et al., 1980; Hoogeveen et al., 2020) and utilized by enterocytes as an energy source at different ratios (Huda-Faujan et al., 2010; Dalile et al., 2019). Acetate is the most abundant SCFA, and it is produced by most microbes, while butyrate and propionate are produced by fewer GI tract bacterial species (Cummings et al., 1987; Morrison and Preston, 2016).

The SCFA regulate various GI functions. For instance, butyrate, acetate, and propionate help to maintain barrier integrity protect from inflammation, and affect mucous production in the GI tract (Dalile et al., 2019). Recently, SCFA are gaining attention for their potential role in the GBA. Studies have found that GLP-1 and PYY secreting EEC, co-expressed SCFA receptors like free fatty acid receptor 2 and 3 (Karaki et al., 2008; Tolhurst et al., 2012), and deletion of these SCFA receptors in EEC in a mouse model has resulted in impaired PYY expression (Samuel et al., 2008) and reduced GLP-1 blood concentration (Tolhurst et al., 2012). Collectively, these findings suggest that SCFA may stimulate the release of these GI hormones that act as an essential mediators of GBA function, as discussed above. SCFA have been shown to promote TPH1 expression in a human carcinoid cell line derived from pancreatic tissues that share functional similarities with EEC, suggesting that SCFA can regulate production of 5-HT by EEC (Reigstad et al., 2015). However, caution must be exercised while translating cell lines result on humans, as these cell divides continuously and may express unique gene patterns that are absent in cells *in vivo* (Kaur and Dufour, 2012). Further evidence of SCFA importance in the GBA comes from a study where butyrate administration by intraperitoneal injection has been shown to attenuate social behavior deficiency in rodents (Kratsman et al., 2016). Butyrate and propionate can also activate tyrosine hydroxylase, the rate-limiting enzyme for catecholamine synthesis (c.f., section “Neurotransmitters”)(Nankova et al., 2014).

Other studies showed that SCFA could also directly influence the GBA. Brain uptake of SCFA was reported following the injection of a mix of ^14^C-SCFA into the carotid artery, which suggests that BBB might be permeable to SCFA (Oldendorf, 1973). SCFA might also directly activate vagal afferents. Luminal perfusion of sodium butyrate into the jejunum of anesthetized male rats evoked vagal efferent nerve responses that were abolished following vagotomy (Lal et al., 2001). Therefore, SCFA can participate in GBA both directly and indirectly; however, further studies are required to understand their role in GBA under physiological conditions.

In the early postnatal period, SCFA production and proportion are expected to change in response to microbial colonization of the GI tract (Midtvedt and Midtvedt, 1992; Norin et al., 2004; Bergström et al., 2014). For instance, exclusively breastfed infants had relatively more acetate in their stools as compared to non-breastfed infants (Bridgman et al., 2017), likely due to the fermentation of oligosaccharides present in human breast-milk by members of the *Bifidobacterium* genus (Azad et al., 2016). The introduction of solid food results in the establishment of different microbial colonizers, which change the SCFA profile in the fecal sample (Differding et al., 2020). However, direct and indirect effects of SCFA production in the early postnatal period on GBA and subsequent consequences for the development of the brain and behaviors are poorly understood.

#### Neurotransmitters

Chemical substances that carry information between neurons are called neurotransmitters. There are about 100 different neurotransmitters produced in the body and each with different functions. Based on chemical composition, neurotransmitters are mainly classified as amino acids and biogenic amines. Functionally, neurotransmitters can be classified as excitatory (increase action potential firing), inhibitory (decrease action potential firing), or modulatory (fine-tune the action of both excitatory and inhibitory neurotransmitters).

Dietary amino acids are precursors for the synthesis of 5-HT, gamma-aminobutyric acid (GABA), norepinephrine, dopamine, and histamine. The synthesis of 5-HT is exclusively from dietary TRP. In contrast, dietary phenylalanine (an essential amino acid) serves as a precursor to tyrosine (a non-essential amino acid), which is essential for the synthesis of norepinephrine and dopamine, and histidine (an essential amino acid) serves as a precursor for histamine (reviewed in Fabisiak et al., 2017; Mittal et al., 2017; Fernstrom and Fernstrom, 2018).

Genes responsible for metabolizing amino acids to neurotransmitters (or precursors of thereof) have been identified in some bacteria, *in vitro*. For instance, *Lactobacillus* and *Klebsiella* spp. possess a histidine decarboxylase gene that converts histidine to produce histamine (Kim et al., 2001; Lucas et al., 2008). *Legionella pneumophila* and *Pseudomonas* spp. have a phenylalanine hydroxylase gene that facilitates the conversion of phenylalanine to tyrosine (precursor of dopamine and norepinephrine), which has been demonstrated *in vitro* (Letendre et al., 1975; Flydal et al., 2012). From the above evidence, it could be speculated that neurotransmitter production by the GI microbes might be modulated by dietary amino acids and contributes to GBA signaling. A list of neurotransmitters and their production by microbial species and their amino acid precursors are shown in Table 1. However, the uptake and metabolism of dietary amino acid by the GI microbiota for neurotransmitter synthesis has not been studied.

**TABLE 1:** Potential neurotransmitters in the gut-brain axis.

** Neurotransmitter**	** Amino acid precursor**	**Microbial species^1^**	** Gastrointestinal tract role**	** Brain role**
Serotonin	Tryptophan	*Escherichia coli* (K-12), *Klebsiella pneumoniae* (Özoğul, 2004; Shishov et al., 2009)	Regulates gastric secretion and motility (Misiewicz et al., 1966)	Mood regulation by decreasing anxiety and stress (Williams et al., 2006)
GABA^2^	Glutamine^3^	*Lactobacillus brevis* and *Bifidobacterium dentium* (Barrett et al., 2012)	Regulates gastric emptying, secretion, and motility (Hyland and Cryan, 2010)	Process sensory information and regulates memory and anxiety (Kalueff and Nutt, 1996)
Dopamine	Phenylalanine	*Escherichia*, and lactic acid-producing bacteria such as *Lactococcus* and *Lactobacilli* spp. (Shishov et al., 2009; Özogul, 2011)	Regulates motility (Li, 2006)	Voluntary movement, induces feeling of pleasure (Juárez Olguín et al., 2016)
Norepinephrine	Phenylalanine	*Escherichia*, *Bacillus*, and *Saccharomyces* spp. (Shishov et al., 2009; Lyte, 2011)	Regulates blood flow (Schwarz et al., 2001)	Motor control, emotion and endocrine modulation (Kobayashi, 2001)
Histamine	Histidine	*Lactobacillus* and *Pediococcus* spp. (Landete et al., 2007; Özogul et al., 2012)	Modulation of motility, enhancement of gastric acid production (Kano et al., 2004; Kim et al., 2011)	Regulates wakefulness, and motivation (Brown et al., 2001; Torrealba et al., 2012)

**^1^The list of bacterial strains is mostly based on in vitro studies and may not be present in the gastrointestinal tract and are provided as examples. ^2^Gamma aminobutyric acid (GABA) is the only inhibitory amino acid neurotransmitter and all others are modulatory biogenic neurotransmitters. ^3^Glutamine is the only non-essential amino acid precursor, whereas all other precursors of neurotransmitters are essential amino acids.**

Some studies report evidence of the metabolism of neurotransmitters by the microbiota. Pathogenic *Escherichia coli* O157:H7 has an increasing growth rate in the presence of norepinephrine and dopamine (Freestone et al., 2002). An extract of peel and pulp of banana, which is rich in neurochemicals (e.g., norepinephrine, dopamine, and 5-HT), has been shown to promote the growth of both pathogenic and non-pathogenic bacteria (Lyte, 1997). The mechanisms by which the GI microbiota can metabolize neurotransmitters *in vivo* are yet to be understood.

There is accumulating evidence *in vivo*, suggesting that the GI microbiota plays a role in modulating the abundance of neurotransmitters. For instance, GF mice have reduced levels of norepinephrine in cecal content (Asano et al., 2012), and of GABA in feces and plasma (Matsumoto et al., 2013). The turnover rate of norepinephrine, dopamine, and 5-HT was higher in the striatum (part of the brain) of GF mice compared with the SPF mice (Heijtz et al., 2011). These reduced levels of neurotransmitters are in line with the altered anxiety-like response in the GF phenotype, suggesting the role of microbiota in the modulation of behavior (Heijtz et al., 2011; Neufeld et al., 2011). However, no studies have yet reported whether the microbiota directly affects the level of neurotransmitters in the body or modulates host production of neurotransmitters. There is also no evidence whether neurotransmitters from the GI tract can cross the BBB to reach the brain. Interestingly, the vagal afferent nerve express receptors for 5-HT, GABA, and dopamine (Egerod et al., 2018), suggesting the possibility of an alternative route for communication between the GI tract and brain. Therefore, GI derived neurotransmitters appear to be a potential mediator of the GBA, and further studies are required to confirm their potential.

In the early postnatal period, histological (e.g., crypt depth) and functional (e.g., enzyme) GI tract changes can result in different rates of amino acid uptake and host neurotransmitter production. The increased relative abundance and diversity of the GI microbiota could also influence neurotransmitter production. For instance, *Bifidobacterium* strains have shown to dominate the GI tract of breastfed infants (Kato et al., 2017; Nagpal et al., 2017; Lawson et al., 2020) and also one of the strain *Bifidobacterium brevis* has shown the ability to produce GABA (Barrett et al., 2012). Change in abundance of different *Bifidobacterium* strains postnatally (Kato et al., 2017) could result in an alteration of the GABA level in the GI tract. Changes in the production of neurotransmitters (type and amount) and their role in the GBA in response to early postnatal developmental remain to be established.

## Concluding Remarks

The early postnatal years of life are marked by rapid developmental changes both in the GI tract and brain. The process of microbial colonization and cognitive development coincide in the first years of life. Sophisticated complex communication systems involving mediators such as VN, GI hormones, cytokines, and the GI-derived metabolites are known to govern the crosstalk between the GI tract and the brain. The establishment of microbes in the GI tract can influence immune (e.g., microglia) and metabolic (e.g., neurotransmitters and TRP metabolites) mediators that ultimately may have an impact on the brain development and behavioral outcomes. Early life foods (breast-milk, formula, and complementary foods) are crucial determinants of GBA mediators in the early postnatal period. Breast-milk could have a potential role in the development of the myelination pattern of VN and the production of hormones in the GI tract, which acts as an essential intermediary between the GI tract and the brain. Overall, the role the GBA mediators during the critical period of development is ill-defined.

It should be noted that many studies relating to the GBA have been carried out on rodent animal models, but considerable differences in developmental patterns of the GI tract and the brain between humans and rodents exist. The use of animal models with more comparable anatomy and physiology (e.g., piglets and primates) to that of humans is desirable to gain a better understanding of the mechanistic pathways of GBA and improve the translation of research to infants. Future research is required to understand whether the expected changes in GBA mediators occur during the critical period of GI tract and brain development and how they can be related to cognitive behavioral outcomes that are the manifestation brain development in infants. For this, longitudinal studies of postnatal life are required. Insights in this area can be targeted via dietary interventions to optimize the communication between the GI tract and the brain to improve cognitive outcomes in infants.

## Author Contributions

AJ, CM, JM, RD, WY, WM, and NR have contributed to the work. AJ conceived and wrote the manuscript. CM and NR helped in structuring the paper and critically reviewed the paper. All other authors advised and critically reviewed versions of the paper. All authors approved the manuscript for publication.

## Conflict of Interest

The authors declare that the research was conducted in the absence of any commercial or financial relationships that could be construed as a potential conflict of interest.

## Abbreviations

5-HT: serotonin
AHR: aryl-hydrocarbon receptor
BBB: blood–brain barrier
EEC: enteroendocrine cells
ENS: enteric nervous system
GABA: gamma-aminobutyric acid
GBA: gut-brain axis
GF: germ-free
GI: gastrointestinal
GLP: glucagon-like peptide
IDO: indoleamine-2,3-dioxygenase
LPS: lipopolysaccharides
PYY: peptide YY
SCFA: short-chain fatty acids
SPF: specific pathogen-free
TPH: tryptophan hydroxylase
TRP: tryptophan
VN: vagus nerve.
